# A Comparative Analysis of the Metabolomic Response of Electron Beam Inactivated *E. coli* O26:H11 and *Salmonella* Typhimurium ATCC 13311

**DOI:** 10.3389/fmicb.2019.00694

**Published:** 2019-04-09

**Authors:** Sohini S. Bhatia, Suresh D. Pillai

**Affiliations:** National Center for Electron Beam Research, International Atomic Energy Agency (IAEA), Collaborating Centre for Electron Beam Technology, Texas A&M University, College Station, TX, United States

**Keywords:** electron beam, inactivation, metabolomics, *E. coli* O26:H11, *Salmonella* Typhimurium

## Abstract

Ionizing radiation such as Electron beam (EB) and gamma irradiation inactivate microbial cells preventing their multiplication. These cells, however, are structurally intact and appear to have residual metabolic activity. We were interested in understanding the metabolic pathways that were still functional in EB-inactivated cells. Therefore, the primary objective of this study was to compare the metabolites accumulating in EB-inactivated pathogens *E. coli* 026:H11 and *S.* Typhimurium immediately after EB inactivation and 24 h post inactivation. Defined aliquots (10^9^ CFU/mL) of *E. coli* O26-H11 (TW 1597) and *S.* Typhimurium (ATCC 13311) suspended in phosphate-buffered saline were exposed to lethal EB doses of 3 kGy and 2 kGy, respectively. Complete inactivation (inability of cells to multiply) was confirmed by traditional plating methods. An untargeted analysis of the primary metabolites accumulating in un-irradiated (control) cells, EB-inactivated cells immediately after irradiation, and EB-inactivated cells that were incubated at room temperature for 24 h post EB inactivation was performed using gas chromatography/mass spectrometry. A total of 349 different metabolites were detected in the EB-inactivated *S*. Typhimurium and *E. coli* O26:H11 cells, out of which, only 50% were identifiable. In *S*. Typhimurium, 98 metabolites were expressed at statistically different concentrations (*P* < 0.05) between the three treatment groups. In *E. coli* O26:H11, 63 metabolites were expressed at statistically different concentrations (*P* < 0.05) between the three treatment groups. In both these pathogens, the β-alanine, alanine, aspartate, and glutamate metabolic pathways were significantly impacted (*P* < 0.01). Furthermore, the metabolomic changes in EB-inactivated cells were amplified significantly after 24 h storage at room temperature in phosphate-buffered saline. These results suggest that EB-inactivated cells are very metabolically active and, therefore, the term Metabolically Active yet Non-culturable is an apt term describing EB-inactivated bacterial cells.

## Introduction

Enteric pathogens such as of Shiga-toxin producing *E. coli* and *Salmonella* Typhimurium account for numerous foodborne outbreaks around the world. *Salmonella* is one of the top 5 foodborne pathogens that cause foodborne illness, while pathogenic *E. coli* are one of the top 4 foodborne pathogens that lead to illnesses and hospitalizations in the United States (CDC, 2018). With significant numbers of outbreaks attributed to fresh produce or minimally processed foods, there is an increasing need for effective non-thermal food processing technologies. Electron beam (EB) processing is a form of ionizing radiation used for food pasteurization in the food industry (Miller, 2005; Hallman, 2011; Lung et al., 2015; Prakash, 2016). The technology is currently used for pasteurizing frozen ground beef in the United States. The EB dose applied to a product is optimized to the intended food product, with the U.S. Food and Drug Administration currently approving doses up to 4 kGy and 7 kGy for lettuce/spinach and for frozen poultry meat, respectively (FDA, *Code of Federal Regulations, title 21, sec. 179.26*). When bacterial cells are exposed to ionizing radiation such as EB, multiple double stranded DNA breaks occur preventing DNA replication and therefore, preventing bacterial multiplication (Urbain, 1986; Tahergorabi et al., 2012; Pillai and Shayanfar, 2017). Complete inactivation of defined titers of bacterial cells by lethal EB doses is achievable based on the knowledge of the target organism’s D_10_ value. Previous studies in our laboratory have shown that when bacterial cells are EB-inactivated, the cell membranes are structurally intact (Jesudhasan et al., 2015). More recent studies in our laboratory have shown that EB-inactivated cells exhibit very defined gene expression patterns and metabolic activity for prolonged periods post the actual EB irradiation (Hieke, 2015; Hieke and Pillai, 2018). Therefore, determining the specific metabolic pathways that are still operating in EB-inactivated cells was of significant interest to us.

The objective of this study was to understand the metabolomic profile of EB-inactivated bacterial pathogens *E. coli* O26:H11 and *S.* Typhimurium ATCC 13311 immediately after EB inactivation and 24 h post EB irradiation and compare these metabolomic profiles to un-irradiated cells. The underlying hypothesis of this study was that even though EB irradiation inactivates bacterial cells, the bacterial cells are capable of maintaining their metabolic pathways even up to 24 h post EB irradiation.

## Materials and Methods

### Bacterial Cell Preparation

*E. coli* O26:H11 (TW 1597) obtained from the USDA-ARS culture collection (USDA-ARS-FFSRU, College Station, TX, United States) and *Salmonella enterica* subsp. e*nterica* serovar Typhimurium (*S*. Typhimurium) (ATCC 13311) were used for this study. Triplicate overnight cultures of each strain were prepared independently by transferring a single colony to Tryptic Soy Broth (TSB) and incubating overnight at 37°C in a shaking water bath. The overnight cultures were washed three times with phosphate-buffered saline (PBS) by centrifugation (4000 ×*g*, 10 min) and suspended in PBS following the last wash. Samples were triple packaged in Whirl-Pak bags (Whirl-Pak, NASCO, Fort Atkinson, WI, United States) (to meet university biosafety protocols) and transported on ice to the EB facility across the campus. Aliquots of the un-irradiated samples were also triple packaged and transported to the EB facility. After processing of irradiated samples, the un-irradiated samples were serially diluted and enumerated on Tryptic Soy Agar (TSA) plates in order to determine the starting titer of the cultures used.

### Electron Beam Inactivation

Electron beam inactivation experiments were performed at the EB facility of the National Center for Electron Beam Research at Texas A&M University in College Station, TX, United States. A 10 MeV, 15 kW linear accelerator delivered the EB doses. Alanine (L-α-alanine) dosimeters were used to confirm the delivered dose. Sample packages were previously dose-mapped to ensure a dose uniformity ratio of one. *E. coli* O26:H11 and *Salmonella* Typhimurium cells were irradiated at target doses of 3 kGy and 2 kGy, respectively. Aliquots of the irradiated bacterial cells were plated on TSA plates immediately after irradiation and 24 h after irradiation to confirm complete inactivation.

### Metabolite Detection and Identification

The primary metabolites in the experimental treatments (namely, un-irradiated control, freshly EB-irradiated cells, and EB-irradiated stored for 24 h at room temperature post irradiation) were detected and analyzed using an untargeted approach using GC-MS at the University of California-Davis metabolomics laboratory. There were three biological replicates for each experimental treatment, and each biological replicate analyzed three times on the GC-MS as technical replicates. Amino acids, hydroxy acids, carbohydrates, sugar acids, sterols, aromatics, nucleosides, amines as well as other co-purifying miscellaneous compounds were extracted, detected, and analyzed using previously published methods (Fiehn et al., 2008, 2010). An Agilent 6890 gas chromatograph (Agilent, Santa Clara, CA, United States) fitted with a 30 m long, 0.25 mm internal diameter Rtx-5Sil MS column with 0.25 μm 95% dimethyl-5% diphenyl polysiloxane film and additional 10 m integrated guard column (Restek, Bellefonte, PA, United States) was used to separate compounds. A volume of 0.5 μl was injected using a Gerstel MPS2 automatic liner exchange system (Mülheim an der Ruhr, Germany). The temperature was increased to 250°C at a rate of 12°C/s. The mobile phase was pure helium (>99.9% purity) at a flow rate of 1 ml/min. The column was held at 50°C for 1 min, ramped to 330°C at a rate of 20°C/min, and held at 330°C for 5 min. Mass spectrometry was performed by a Leco Pegasus IV time of flight mass spectrometer (St. Joseph, MI, United States) with a -70 eV ionization energy, 1800 V detector voltage, 230°C transfer line temperature, and 250°C ion source temperature. Mass spectra were acquired with unit mass resolution at 17 spectra/s from 80 to 500 Da.

The raw data files were pre-processed directly after data acquisition and all entries were processed by the metabolomics BinBase database. Identified metabolites were reported if present within at least 50% of the total samples. Data was reported as peak heights for the quantification ion (*m*/*z* value) at the specific RI. Peak heights were used because peak heights are more precise than peak areas at quantifying metabolites found in low concentrations. Any metabolite with more than one peak, were summed and reported as a single value. The raw data was transformed and normalized by dividing each raw metabolite value by the sum intensities of all known (excluding unknown) metabolites in that sample. This value was then multiplied by a constant factor to obtain whole numbers. This transformation was done in order to normalize the data to total known metabolite content, disregarding unknowns that could potentially contain artifact peaks or chemical contaminants.

### Statistical Analysis

A statistics-based analysis of the primary identified and unknown metabolites was performed using MetaboAnalyst 4.0, a web-based metabolomics processing tool^1^. The data was normalized using log transformed and Pareto scaling feature of MetaboAnalyst. A one-way ANOVA using Tukey’s HSD was used to determine significant features. Partial Least Squares - Discriminant Analysis (PLS-DA) was used to examine differences between the treatment groups. For pathway analysis using MetaboAnalyst 4.0, the mean peak value was considered to analogous to mean metabolite concentration. Global test and relative-betweenness centrality algorithms were used for pathway enrichment and pathway topology, respectively. Furthermore, all *P* values were adjusted for the False Discovery Rate (FDR). The FDR was subsequently used for all significance tests. *S.* Typhimurium and *E. coli* O26:H11 samples were analyzed separately. Given the close taxonomic relatedness between *S*. Typhimurium and *E. coli*, and the lack of publicly available metabolite pathway databases for *S.* Typhimurium, the metabolite library of *E. coli* K-12 MG1655 was used as the pathway library for both *S.* Typhimurium and *E. coli* pathway analysis.

## Results

### Confirmation of Bacterial Inactivation

The starting titer of *E. coli* and *S.* Typhimurium was 9.36 ± 0.02 log CFU/ml and 9.24 ± 0.20 log CFU/ml, respectively. The *S*. Typhimurium samples received a measured dose of 1.94 kGy and *E. coli* samples received a measured dose of 3.02 kGy. No growth was detected in any of the irradiated samples, confirming that these EB doses achieved complete inactivation of the experimental samples.

### Metabolomic Analysis

A total of 349 metabolites were detected, out of which only 175 were identifiable (Supplementary Data 1, 2). Overall, only 50% of the detected metabolites in *E. coli* O26:H11 and *S*. Typhimurium were identifiable using publicly available databases such as KEGG and PubChem. In *E. coli* O26:H11, 63 metabolites were expressed at different concentrations (*P* < 0.05) between the three *E. coli* experimental treatment groups (Supplementary Data 3). Ninety eight (98) metabolites were found in significantly different concentrations (*P* < 0.05) between the *S*. Typhimurium treatment groups (Supplementary Data 4).

### Metabolic Pathway Analysis

**FIGURE 1:**
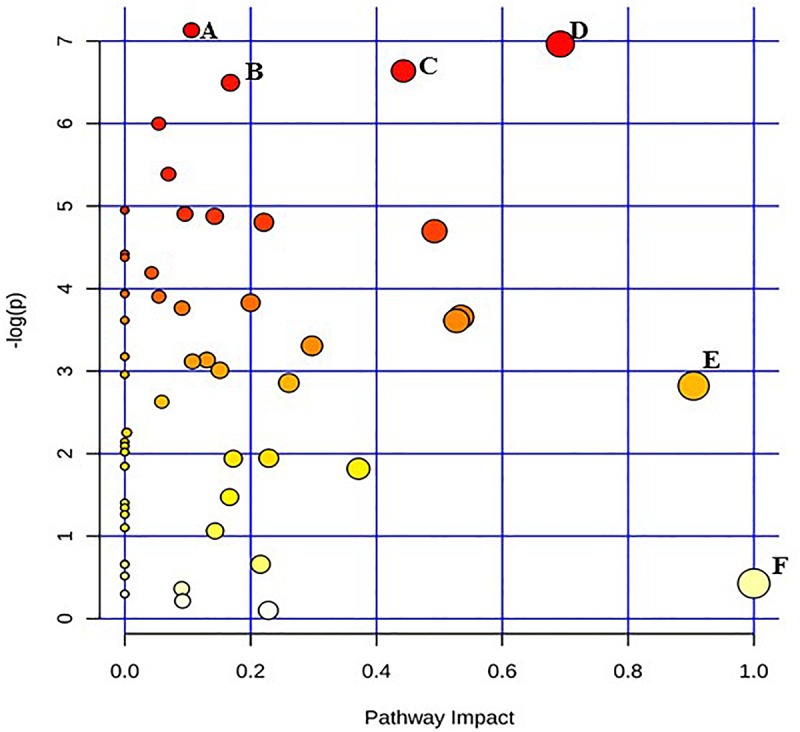
Pathway analysis highlighting important pathways of *E. coli* O26:H11 immediately after irradiation (EB 0 h) compared to un-irradiated cells (0 kGy Control). The *Y* axis represents metabolic pathways containing metabolites that are significantly different between treatment groups. They *X* axis represents the impact these significantly changed metabolites have on the overall pathway based on their position within the pathway. A: Pentose and glucuronate interconversions; B: Pantothenate and CoA biosynthesis; C: Starch and sucrose metabolism; D: Beta alanine; E: Alanine, aspartate, and glutamate metabolism; F: Inositol phosphate metabolism.

**FIGURE 2:**
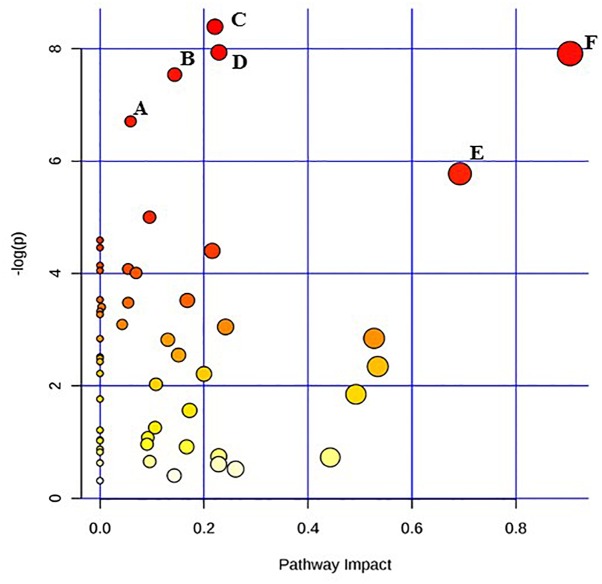
Pathway analysis highlighting important pathways of *E. coli* O26:H11 24 h post EB exposure (EB 24 h) compared to freshly irradiated cell (EB 0 h). The *Y* axis represents metabolic pathways containing metabolites that are significantly different between treatment groups. They *X* axis represents the impact these significantly changed metabolites have on the overall pathway based on their position within the pathway. A: Butanoate metabolism; B: Nicotinate and nicotinamide metabolism; C: Cysteine and methionine metabolism; D: Citrate cycle (TCA cycle); E: beta-Alanine metabolism; F: Alanine, aspartate and glutamate metabolism.

**FIGURE 3:**
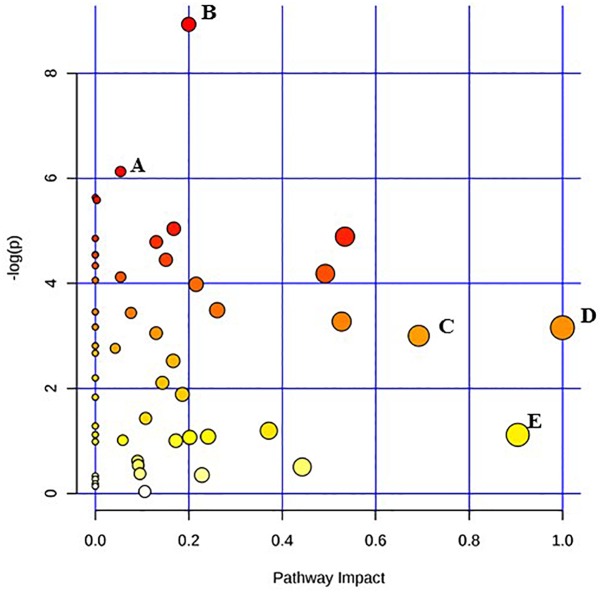
Pathway analysis highlighting important pathways of *S.* Typhimurium immediately after irradiation (EB 0 h) compared to un-irradiated cells (0 kGy Control). The *Y* axis represents metabolic pathways containing metabolites that are significantly different between treatment groups. They *X* axis represents the impact these significantly changed metabolites have on the overall pathway based on their position within the pathway. A: Propanoate metabolism; B: Tryptophan metabolism; C: beta-Alanine metabolism; D: Inositol phosphate metabolism; E: Alanine, aspartate and glutamate metabolism.

**FIGURE 4:**
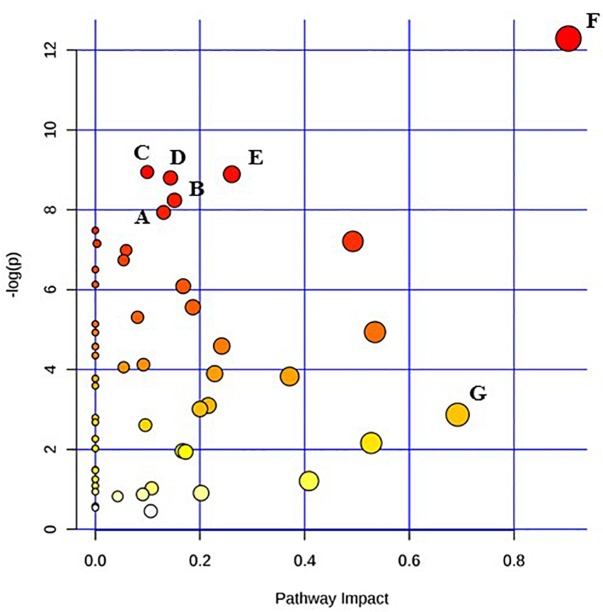
Pathway analysis highlighting important pathways of *S.* Typhimurium 24 h post EB (EB 24 h) exposure compared to freshly irradiated cell (EB 0 h). The *Y* axis represents metabolic pathways containing metabolites that are significantly different between treatment groups. They *X* axis represents the impact these significantly changed metabolites have on the overall pathway based on their position within the pathway. A: Aminoacyl-tRNA biosynthesis; B: Glyoxylate and dicarboxylate metabolism; C: Cysteine and methionine metabolism; D: Nicotinate and nicotinamide metabolism; E: Glycerolipid metabolism; F: Alanine, aspartate and glutamate metabolism; G: beta-Alanine metabolism.

Given the current technical challenges in analyzing more than two metabolic pathways simultaneously, two separate metabolic pathway analyses were conducted for each of the two target pathogens. One comparison was between the un-irradiated control cells and the freshly irradiated cells. The other comparison was between freshly irradiated cells and irradiated cells that were stored at room temperature for 24 h. Figure 1 shows the results of the analysis between un-irradiated *E. coli* O26:H11 cells and freshly irradiated cells (control vs. 0 h). Figure 2 shows the results of the analysis of the freshly irradiated *E. coli* O26:H11 cells and the irradiated cells stored for 24 h (0 h vs. 24 h). Eleven pathways were significantly different (*P* < 0.05) between the *E. coli* O26:H11 control and 0 h groups (Supplementary Data 5), while 6 pathways were significantly different (*P* < 0.05) between the 0 h and 24 h *E. coli* O26:H11 (Supplementary Data 6). Figure 3 shows the results of the pathway analysis of un-irradiated *S.* Typhimurium and freshly irradiated *S.* Typhimurium (control vs. 0 h). Figure 4 shows the results from the comparison of the metabolic pathways from the freshly irradiated *S*. Typhimurium and EB-irradiated *S.* Typhimurium stored for 24 h (0 h vs. 24 h). Four pathways were significantly different (*P* < 0.05) between the *S.* Typhimurium control and 0 h groups (Supplementary Data 7), while 27 pathways were significantly different (*P* < 0.05) between 0 and 24 h *S.* Typhimurium.(Supplementary Data 8).

## Discussion

**FIGURE 5:**
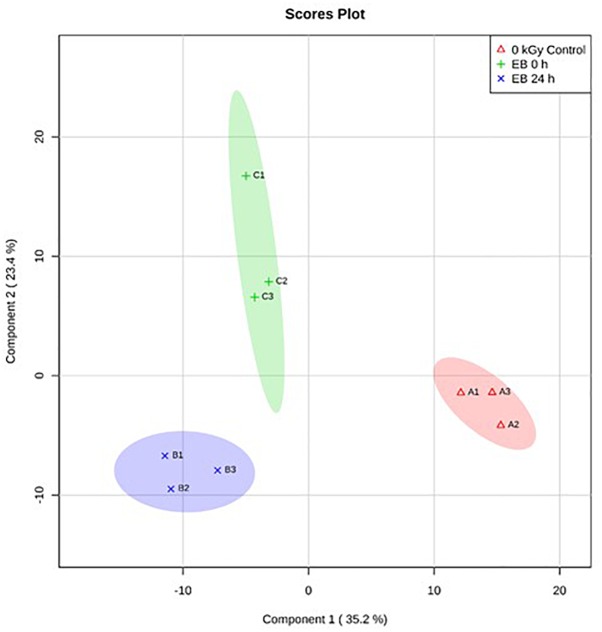
Partial Least Square-Discriminant Analysis (PLS-DA) scores plot showing differences between the un-irradiated (0 kGy Control), irradiated (EB 0 h), and 24 h post irradiation (EB 24 h) *E. coli* O26:H11.

**FIGURE 6:**
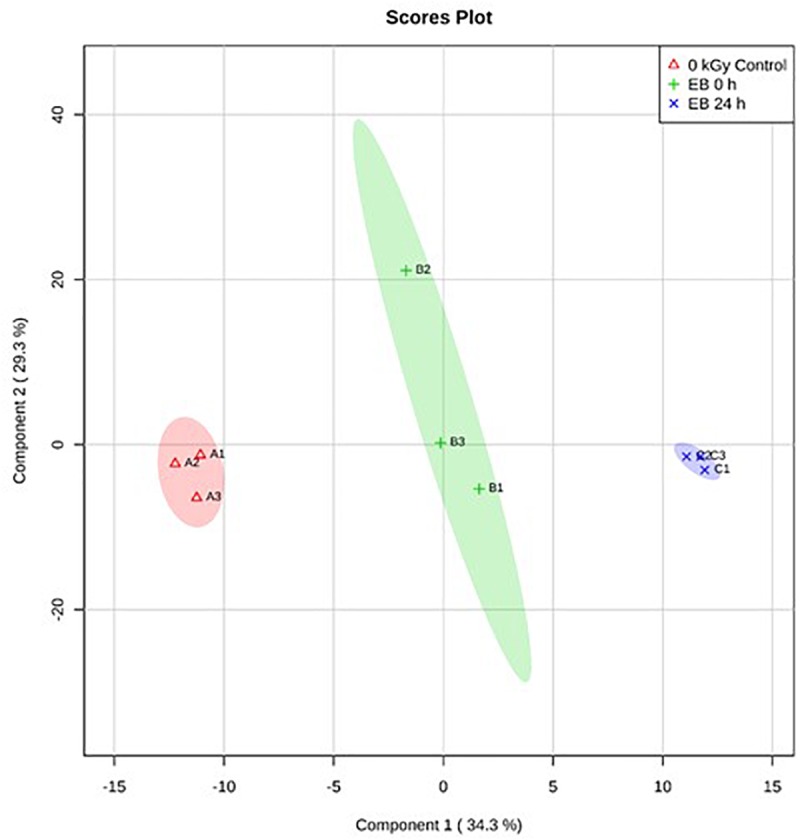
Partial Least Square-Discriminant Analysis (PLS-DA) scores plot showing differences between the un-irradiated (0 kGy Control), irradiated (EB 0 h), and 24 h post irradiation (EB 24 h) *S.* Typhimurium.

Based on PLS-DA, there were clear differences between the *E. coli O26:H11* treatment groups, with 35.2% of the variance explained by component 1 (Figure 5). In *S.* Typhimurium, there were also clear differences between treatment groups, with 34.4% of the variance explained by component 1 (Figure 6). The PLS-DA scores plots for both target pathogens show tightly clumped biological replicates of the control and 24 h post EB cells. The biological replicates of freshly irradiated cells were more spread out, indicating that there was more variance in the metabolites contributing to the key features of this treatment group. The high variance between the biological replicates of freshly irradiated cells, seen with both pathogens suggests that the immediate response to ionizing radiation is quite variable. Multiple pathways may be expressed in order to protect and repair the bacteria after exposure to lethal EB.

Pathway enrichment analysis is based on quantitative analysis that directly utilizes metabolite concentration values. This type of analysis allows for detecting subtle differences in metabolites involved in the same biological pathway (Xia and Wishart, 2016; Chong et al., 2018). A global test algorithm was used to test for patterns of differentially expressed metabolites between treatment groups (Goeman et al., 2004; Hendrickx et al., 2012). Pathway topology analysis was performed to identify the impact a particular metabolite had on a particular metabolic pathway. Metabolic pathways that were considered the “most significant” were those that had a high impact value above 0.6 and/or statistical significance above –log(p) 6. Table 1 summarizes the most impacted pathways in *E. coli* O26:H11 and *S*. Typhimurium ATCC 13311 after EB irradiation.

**Table 1:** Most significantly impacted metabolic pathways in *E. coli* O26:H11 cells and *S.* Typhimurium ATCC 13311 when exposed to a lethal EB dose as a function of time post EB exposure.

Comparing 0 kGy and 3 kGy exposed *E. coli* O26:H11 cells (0 kGy Control and EB 0 h)	Comparing 3 kGy exposed *E. coli* O26:H11 cells immediately after exposure and 24 h post EB exposure (EB 0 h and EB 24 h)
Pentose and glucuronate interconversions	Butanoate metabolism
Pantothenate and CoA biosynthesis	Nicotinate and nicotinamide metabolism
Starch and sucrose metabolism	Cysteine and methionine metabolism
Beta-alanine metabolism	Beta-Alanine metabolism
Alanine, aspartate, and glutamate metabolism	Alanine, aspartate and glutamate metabolism
Inositol phosphate metabolism	Citrate cycle (TCA cycle)
	
**Comparing 0 kGy and 2 kGy *S*. Typhimurium ATCC 13311 exposed cells (0 kGy Control and EB 0 h)**	**Comparing 2 kGy exposed *S*. Typhimurium ATCC 13311 cells immediately after exposure and 24 h post EB exposure (EB 0 h and EB 24 h)**
Propanoate metabolism	Propanoate metabolism
Tryptophan metabolism	Phenylalanine, tyrosine and tryptophan biosynthesis
Beta-Alanine metabolism	Beta-Alanine metabolism
Inositol phosphate metabolism	Cysteine and methionine metabolism
Alanine, aspartate, and glutamate metabolism	Alanine, aspartate, and glutamate metabolism
	Butanoate metabolism
	Nicotinate and nicotinamide metabolism
	Glycerolipid metabolism
	Benzoate degradation via CoA ligation
	Arginine and proline metabolism
	Phenylalanine metabolism
	Tyrosine metabolism
	Pantothenate and CoA biosynthesis
	Aminoacyl–tRNA biosynthesis
	Glyoxalate and dicarboxylate metabolism


Irrespective of whether it is *E. coli* O26:H11 or *S*. Typhimurium, the primary metabolites in these cells are very distinct depending on whether they are un-irradiated or freshly irradiated or stored for 24 h post EB irradiation (Figure 1, 2). The response of bacterial cells to stressors such as acid, temperature, and ionizing radiation have been extensively studied (Foster, 1991; Arsène et al., 2000; Castanié-Cornet et al., 2010; Daly, 2012; Pin et al., 2012; Swenson et al., 2012; Castillo and Smith, 2017; Villa et al., 2017). Recently, we have shown that *E. coli* O26:H11 when exposed to acid stress (pH 3.6), the key differentially expressed pathways were peptidoglycan biosynthesis, purine metabolism, D-Glutamine/D-glutamate metabolism, nitrogen metabolism, unsaturated fatty acid biosynthesis, and inositol phosphate metabolism (Shayanfar et al., 2018 *J. Appl. Microbial*. *submitted manuscript*). The differentially expressed pathways post EB irradiation match these pathways very closely except for peptidoglycan synthesis (Table 1). It is noteworthy that the cell wall associated peptidoglycan pathway is not affected during EB irradiation and this is reflected in our findings that the cellular structure is unaffected during EB irradiation (Jesudhasan et al., 2015). Similarity in the perturbations of the other pathways between acid stress exposure and EB irradiation exposure suggests that these pathways are part of the general stress response in bacterial cells. Across all treatments and considering both *E. coli* O26:H11 and *S*. Typhimurium, the β-alanine and alanine, aspartate, and glutamate pathways were significantly impacted (Table 1). Increase in amino acid synthesis is a stress response commonly seen in *E. coli.* Stapleton reported that irradiated *E. coli* cells require amino acids such as glutamic and aspartic acid for recovery from radiation injury (Stapleton, 1955). Thus, it is indicative from these studies that the irradiated cells are attempting to synthesize these critically important amino acids. Increased levels of alanine, aspartate, and glutamate have also been observed in the cold stress response (Jozefczuk et al., 2010). Since ionizing radiation at 2 kGy and 3 kGy does not significantly impact proteins, the increase of amino acids could also be a result of intentional protein breakdown (Mandelstam, 1963; Willetts, 1967; Tsakalidou and Papadimitriou, 2011).

Immediately after EB treatment, *E. coli* had increased β-alanine and Pantothenate metabolism. β -alanine is a direct precursor for pantothenate synthesis, explaining why the two are seen together immediately after irradiation in *E. coli* and after 24 h in *Salmonella* (Schneider et al., 2004). Pantothenate has been reported to be involved in cell wall and cell membrane biosynthesis (Toennies et al., 1966). Therefore the enhancement of the pantothenate metabolism suggests that the EB irradiated cells are possibly attempting to repair possible damages to the cellular membrane and walls that are yet undetectable. Both species showed significant increases in inositol phosphate metabolism immediately after irradiation, with the significant increase in the concentration of myo-inositol showing no change after 24 h. This is noteworthy as myo-inositol does not accumulate in bacteria; rather, it is converted to soluble phosphate esters (Roberts, 2006). While there have been very few studies conducted on the role of these compounds in prokaryotes, they are important signaling molecules in yeast cells and other eukaryotes, and exploration into their metabolic function would be of significant biologic importance (Wilson et al., 2013). Significant changes in metabolic activity occurred in both *E. coli* O26:H11 and *S.* Typhimurium even 24 h post exposure to lethal doses of EB. While not immediately triggered, there was a significant increase in the citrate cycle in *E. coli* 24 h after exposure to EB. The TCA cycle is one of the most important metabolic pathways in all oxidative organisms driving not only ATP generation, but also the defense against reactive oxygen species (Shimizu, 2013). Specifically, α-ketoglutarate plays a very important role in the detoxification of H_2_O_2_ and O_2_^-^ (Mailloux et al., 2007). Interestingly, α-ketoglutarate levels initially increased after irradiation, but after 24 h, they decreased. This suggests that while there is an intial spike in concentration, over the course of 24 h, the cell either cannot or does not need to maintain elevated levels of this metabolite. In both species, succinic acid levels were increased immediately after irradiation, and continued to increase 24 h later. Accumulation of succinic acid has been previously observed and is a biomarker of oxidative stress (Mailloux et al., 2007). Furthermore, the accumulation of succinic acid is also used as a signaling mechanism in oxidatively stresed eukaryotic cells (Tretter et al., 2016). *S.* Typhimurium appears to have a more delayed metabolic reaction to EB, compared to *E. coli* O26:H11. Many of the pathways were impacted only 24 h after exposure to EB (Table 1). These include multiple pathways devoted to amino acid metabolism, namely the biosynthesis and metabolism of cysteine, methionine, alanine, aspartate, glutamate, arginine, proline, phenylalanine, tyrosine, and tryptophan. This is similar to the response seen in *E. coli* O26:H11, but on a much larger scope. Previous studies have also demonstrated distinct differences in the rate at which these two pathogens react to sudden changes (Sargo et al., 2015; Burgess et al., 2016). A major drawback of untargeted primary metabolomic analysis is the large presence of unknown metabolites. In this dataset, 50% of the metabolites detected were unknown. Many of the unknown metabolites had significantly different concentrations, and could potentially be key biomarkers of irradiation exposure. However, because their identity was unknown, these metabolites were excluded from the pathway analysis.

Overall, the results of this study clearly show that metabolites in both *E. coli* O26:H11 and *S*. Typhimurium exhibit fluxes in concentration even 24 h post EB irradiation exposure. The origin of these fluxes needs to be clearly understood. Since we did not compare the metabolites of un-irradiated cells stored for 24 h, we did not account for the possible likelihood that auto-oxidation of metabolites occurring. Are changes in metabolite concentration a function of the residual gene expression that we observed in these cells (Hieke, 2015)? Or are these metabolite concentrations changing due to purely abiotic effects within the cells? The continued gene expression coupled with our previous studies showing increased H+ exchanges within the cells (based on alamar blue staining) suggests that even after 24 h post lethal EB irradiation, bacterial cells are metabolically active and still attempting to repair their damage (Praveen, 2014). Detailed studies are needed to identify whether the presence of certain unique metabolites in bacterial cells can be used as biomarkers of exposure to ionizing radiation exposure.

## Author Contributions

SB and SP designed the experiments. SB performed the laboratory experiments. SB and SP were involved in preparing the manuscript for publication.

## Conflict of Interest Statement

The authors declare that the research was conducted in the absence of any commercial or financial relationships that could be construed as a potential conflict of interest.

## Footnotes

1 http://www.metaboanalyst.ca/
