# Supplementary material for: A Comparative Analysis of the Metabolomic Response of Electron Beam Inactivated E. coli O26:H11 and Salmonella Typhimurium ATCC 13311
Source: Front Microbiol. 2019 Apr 9;10:694. doi: 10.3389/fmicb.2019.00694 (PMC6465604; doi:10.3389/fmicb.2019.00694)
Supplement: Supplementary file 1 [file Data_Sheet_1.pdf]

Supplementary Data 1. Average Metabolite Concentration of Un-irradiated (0 kGy Control), Freshly Irradiatied (EB 0 h), 24 Hour Post-Irradiation (EB 24 h) *E. coli* O26:H11

Metabolite	Average Metabolite Concentration		
	0 kGy Control	EB 0 h	EB 24 h
L-Threo-2-pentulose	1087	993	1803
D-Xylose	2685	13713	14167
D-Xylono-1,5-lactone	2628	1393	1797
Xanthosine	336	522	4242
Xanthine	2788	5518	7355
L-Valine	154300	84687	304568
Urocanic acid	116	155	166
Uridine 5'-monophosphate	2133	1123	760
Uridine	7694	9319	5190
Uracil	98576	116852	267797
Tyrosol	61	50	68
L-Tyrosine	57352	28617	32979
L-Tryptophan	8473	5856	10038
Trehalose	2775	2022	1400
Thymine	2444	9553	30700
5-Thymidylic acid	317	2121	1687
Threonine	7045	5482	7418
Lignocerane	1549	3766	1215
2,3-Dihydroxybutanedioic acid	49	736	844
Sucrose	646	1992	15547
Succinic acid	6840	9989	43369
Stearic acid	1176491	1386187	1178966
Spermidine	376239	195561	325089
Serine	9841	9203	8017
2-Hydroxybenzaldehyde	429	426	1172
D-Ribulose 5-phosphate	822	924	576
Ribose-5-phosphate	1184	1107	1196
Ribose	41206	38062	51555
Ribonic acid	276	341	418
Raffinose	77	52	46
Pyrophosphate	130598	80031	64453
Putrescine	851406	552839	644559
Pseudouridine	1228	1112	4526
Proline	24197	13850	6921
Pipecolinic acid	904	385	811
Pinitol	27479	6064	7382
Hydroxyphenyllactic acid	514	711	4308
Phthalic acid	1995	4075	4915
Phosphoethanolamine	11420	14843	16456
Phosphoenolpyruvate	3153	3371	1837
Phosphate	298265	276712	272488

Average Concentration

High

Medium

Low

Phenylpyruvate	2616	5044	10505
Phenylethylamine	11317	8696	8580
Phenylalanine	28256	13950	22880
Pentadecanoic acid	6339	18118	29679
Pelargonic acid	3006	8073	2897
Oxalureate	638	12650	18916
Pantothenic acid	1987	1177	2297
Palmitic acid	149712	246634	149418
Pyroglutamic acid	143503	139006	60362
Oxalic acid	212	2995	2686
Orotic acid	6573	180	159
Ornithine	26362	9495	12031
Oleic acid	295	481	268
Octadecanol	343	576	273
Noradrenaline	188	1635	807
Nonadecanoic acid	1311	1898	1214
Nicotinic acid	2353	2020	18772
Nicotinamide	16327	15380	7387
N-Acetylputrescine	802201	1877	370540
N-Acetylornithine	1309	15302	18985
Acetylglycine	330	913	672
N-Acetyl-D-galactosamine	172	780	626
N-Acetylaspartic acid	328	628	708
Myristic acid	2698	4310	3307
Myo-Inositol	361	544	356
Methionine sulfoxide	24615	7937	15335
Methionine	4738	2055	3440
Maltotriose	1157	919	246
Maltose	13339	10670	3973
Malonic acid	95	1986	2407
Malic acid	2379	3735	7444
Lyxose	1973	929	1472
Lysine	137263	88028	78755
Leucine	51406	20819	49772
Lauric acid	4806	9350	6463
Lactulose	552	723	700
Lactic acid	2086	7256	3480
Prostaglandin F2a	2585	968	917
D-Threitol	42	77	643
Isoribose	281	175	1663
Isoleucine	92727	47881	99635
Inosine	243	201	5021
Indole-3-lactate	145	286	1478
indole-3-acetate	1395	897	6069
Hypoxanthine	1803	3666	191369
Hydroxylamine	30193	28787	31749
Homoserine	2591	1971	3762

**Histidine**	17280	6607	13536
**Glucose 6-phosphate**	941	353	81
**Heptadecanoic acid**	7142	12693	8095
**Guanosine**	7295	5974	7594
**Guanine**	11705	5544	8924
**Glycolic acid**	1593	6157	14806
**Glycine**	14056	7501	9737
**DL-Glycerol 1-phosphate**	8867	9794	10808
**Galactosylglycerol**	2870	1116	1765
**Glyceric acid**	3919	7089	20577
**Glutathione**	2017	1455	282
**Glutaric acid**	670	428	4034
**Glutamine**	1327	479	1553
**Glutamic acid**	82467	39991	22770
**Glucose-6-phosphate**	1471	984	42
**Glucose-1-phosphate**	1858	1750	2464
**Glucose**	1377	1882	1265
**Galactonic acid**	477	346	533
**Galactinol**	174	123	156
**Fumaric acid**	5180	6398	16501
**Fucose**	8722	7748	11768
**Fructose-6-phosphate**	759	356	126
**Fructose**	293	2141	1411
**Ethanolamine**	48490	33181	288279
**Dodecanol**	434	989	448
**Dehydroascorbic acid**	276	4235	4294
**Dehydroabietate**	3634	2680	2651
**Cytosine**	864	1137	1455
**Cytidine monophosphate**	1934	3226	1741
**Cysteinylglycine**	6270	1875	2390
**Cysteine**	8087	570	1701
**Conduritol-beta-epoxide**	11462	1818	1606
**Citrulline**	4827	2522	2416
**Citric acid**	12168	26915	18047
**Citramalic acid**	225	877	760
**Citraconic acid**	4621	10041	20075
**Cholesterol**	343	734	1067
**Cellobiose**	2442	1966	797
**Capric acid**	233	878	348
**2-Pyrrolidinone**	1620	1644	1349
**Beta-Hydroxymyristic acid**	156	120	223
**Beta-Glycerophosphoric acid**	344	330	367
**Gentiobiose**	335	73	39
**Beta-Alanine**	323	4399	6258
**Benzoic acid**	1020	1534	976
**Behenic acid**	3636	4930	5074
**Aspartic acid**	34013	23804	11667

Arachidic acid	13176	21259	13067
Aminomalonate	414	173	324
Alpha-Ketoglutarate	458	994	293
Alpha-Aminoadipic acid	120	183	605
Alanine-?-Alanine	91995	31179	23405
D-Alanyl-D-alanine	2577	9888	701
Alanine	60446	41338	75911
Adipic acid	1432	2517	1176
Adenosine monophosphate	22851	15192	23656
Adenosine	28222	28424	30923
Adenine	95959	81243	142314
Acetophenone	2858	3899	2671
7-Methylguanine	111	110	652
Quinovose	1564	31547	45045
Glucitol, 6-deoxy-	2029	1218	1406
5-Methoxytryptamine	8531	8416	7454
5'-Methylthioadenosine	2838	4018	2845
5-Aminovaleric acid	3476	1457	9884
4-Hydroxyphenylacetic acid	5781	2979	21905
4-Hydroxybutyric acid	677	99	804
4-Hydroxybenzoate	748	469	2137
4-Aminobutanoate	25328	6966	2145
3-Phosphoglycerate	17465	15615	6098
3-Phenyllactic acid	3012	2838	24507
3-Hydroxybutyric acid	1065	3476	2746
3'-Adenylic acid	7167	3851	3715
Propanoic acid	503	282	888
2-Ketoisocaproic acid	7210	22715	54290
2-Ketoadipic acid	2295	3289	3435
2-Hydroxyvaleric acid	1759	3888	4042
2-Hydroxyhexanoic acid	3393	1171	12003
D-2-Hydroxyglutaric acid	1319	723	2957
2,4-Dihydroxybutanoic acid	3181	3581	4109
2,5-Dihydroxypyrazine	12955	4542	3890
2,4-Diaminobutyric acid	104	3146	2351
4-Deoxyerythronic acid	47	454	805
1-Monostearin	357	442	400
1-Monopalmitin	1836	1043	1253
1-Deoxyerythritol	109358	35432	5858
1,3-Diaminopropane	1922	1793	2104
704730	710	292	983
160962	2863	5308	2475
160842	5533	2619	7133
159824	2389	2760	1788
146957	3337	2972	3097
146262	278	316	913
146042	55724	9494	10451

145865	508	794	4611
134760	1388	32354	45153
134752	48	260	349
134122	272	395	587
133242	2118	4134	3164
132976	2205	1366	513
131620	78259	73198	71453
130797	1432	4397	4123
130396	805	761	630
129313	94	52	73
127277	3307	3361	4913
125786	256	2153	834
124903	8466	14662	8380
124568	4782	885	1252
123989	83	534	592
121002	1404	1736	1540
120789	1292	1371	1347
119066	1080	1996	1208
113700	2529	907	4253
112264	26992	26606	32162
111826	3540	6703	3115
111057	694	480	356
110359	511	679	1351
110346	382	1011	1071
110265	205	1104	1989
110131	1556	769	1421
108309	479	341	1622
106742	180167	266625	220710
104906	3677	5502	4408
104022	5706	2213	2602
103857	69	495	265
103138	5819	4218	6381
103102	25569	25581	22518
102232	3388	1569	1779
100723	6074	361	4965
88786	4479	1749	1144
88502	3156	4956	3709
88046	4439	2311	1754
87947	78	60	66
87312	1075	3813	691
87282	3434	2724	12689
84565	1630	1849	622
84209	8347	8782	7402
66261	74	2104	1147
48608	68	1444	1401
47420	4657	2933	6953
47170	841	1349	594

**46357**	2660	10006	13162
**46128**	694	1077	624
**41989**	76	661	674
**41811**	851	1016	797
**41808**	838	1217	1187
**33999**	69	297	379
**32148**	529	729	3235
**31460**	1147	2014	1031
**31408**	1287	1986	1361
**31362**	4132	8221	3384
**31359**	1406	2801	1768
**31285**	3579	4742	10527
**26062**	155	333	149
**21885**	1176	2227	1128
**21683**	2697	2760	3462
**21666**	3746	3591	1662
**21665**	1737	4265	2027
**21664**	5167	4981	3338
**21511**	862	1210	2118
**20903**	6504	3321	5572
**20330**	2590	5499	2008
**20282**	2898	980	2475
**18588**	1129	916	1152
**18485**	3670	5090	2447
**18266**	915	394	357
**18248**	290	87	707
**18225**	864	856	923
**18177**	2879	977	2252
**17962**	3472	6650	2988
**17830**	1116	479	572
**17775**	42	684	567
**17651**	440	833	380
**17463**	146	140	114
**17437**	2248	1076	1681
**17245**	1195	1584	1272
**17068**	667	593	731
**17002**	1162	740	1212
**14703**	1414	2393	3876
**14697**	1821	635	424
**10176**	122	170	472
**9320**	7218	9237	4324
**7408**	877	2990	2576
**7403**	112	4367	4127
**5691**	1709	2761	12472
**5523**	2450	4726	2016
**5346**	4978	8195	4846
**4945**	757	850	832

4937	970	1633	971
4850	28	912	1159
4712	809	1702	1735
4550	964	1138	746
4265	3174	6405	2860
4264	3794	8133	2718
4263	4529	8899	4139
3188	1620	3653	3509
3122	18608	11849	9076
2847	347	838	316
2706	1184	2803	1650
2543	1596	546	921
2503	2354	1147	589
2438	11932	6058	11144
2262	4969	7696	7518
2242	10151	7390	9490
2233	3224	2367	1786
2042	2540	3648	8875
2039	1868	2366	3258
2031	20382	7438	8944
2030	1353	1624	1530
2028	685	378	470
2017	640	453	1899
2001	2397	685	269
1996	332	3450	3333
1981	1637	1458	2141
1970	2377	860	768
1969	383	437	472
1941	743	572	764
1912	1393	2599	1359
1878	3584	6819	4550
1875	4244	6518	4605
1872	5281	7023	5483
1852	418	341	1216
1826	1044	313	1182
1815	1591	5839	2732
1812	1339	838	650
1809	1876	1510	1554
1806	5359	985	2196
1805	2648	3101	1095
1803	351	635	714
1799	8939	72153	85972
1760	2449	837	500
1753	4114	5572	6515
1751	6227	8761	6493
1746	893	463	452
1744	724	1027	684

**1737**	3615	802	1381
**1735**	1959	666	965
**1725**	3132	4501	2837
**1721**	1789	1053	1169
**1719**	162	84	1036
**1717**	20237	15429	13994
**1713**	2566	782	718
**1708**	3167	1212	1236
**1702**	6650	1260	1859
**1701**	879	172	610
**1696**	344	267	346
**1675**	7346	551	236
**1673**	1991	929	1508
**1666**	1242	1368	2382
**1661**	2712517	1715809	2601839
**1064**	2109	4126	2276
**816**	5414	4083	7335
**453**	27407	21061	28284
**443**	10205	10148	8663
**307**	9950	28000	16211
**257**	1579	2363	1332
**168**	1395	1078	1258
**137**	2714	8944	4683
**134**	14416	9573	7818
**110**	2124	2837	4747
**47**	7671	13929	8800
