# Supplementary material for: A Comparative Analysis of the Metabolomic Response of Electron Beam Inactivated E. coli O26:H11 and Salmonella Typhimurium ATCC 13311
Source: Front Microbiol. 2019 Apr 9;10:694. doi: 10.3389/fmicb.2019.00694 (PMC6465604; doi:10.3389/fmicb.2019.00694)
Supplement: Supplementary file 2 [file Data_Sheet_2.pdf]

Supplementary Data 2. Average Metabolite Concentration of Un-irradiated (0 kGy Control), Freshly Irradiatied (EB 0 h), 24 Hour Post-Irradiation (EB 24 h) *S.* Typhimurium

Metabolite	Average Metabolite Concentration		
	0 kGy Control	EB 0 h	EB 24 h
L-Threo-2-pentulose	2805	2275	2590
D-Xylose	6847	1	7173
D-Xylono-1,5-lactone	3108	2484	1911
Xanthosine	393	198	1349
Xanthine	96	36	289
L-Valine	107503	42885	159849
Urocanic acid	590	357	955
Uridine 5'-monophosphate	713	486	416
Uridine	30206	38188	34662
Uracil	250447	194823	350854
Tyrosol	117	142	1890
L-Tyrosine	42433	15391	27236
L-Tryptophan	16733	4535	9892
Trehalose	738	362	134
Thymine	2678	4320	18744
5-Thymidylic acid	444	385	798
Threonine	5901	2834	3184
Lignocerane	1593	1370	1880
2,3-Dihydroxybutanedioic acid	61	447	488
Sucrose	4171	79580	102235
Succinic acid	1691	2613	21468
Stearic acid	1164487	821496	1352128
Spermidine	290154	192745	151005
Serine	7627	4411	5298
2-Hydroxybenzaldehyde	268	226	4056
D-Ribulose 5-phosphate	381	361	209
Ribose-5-phosphate	655	887	551
Ribose	121047	105492	88376
Ribonic acid	171	344	254
Raffinose	727	645	507
Pyrophosphate	96915	94100	45286
Putrescine	1539597	1118267	1238998
Pseudouridine	902	795	3219
Proline	10519	6136	4101
Pipecolinic acid	202	132	181
Pinitol	5813	1937	1579
Hydroxyphenyllactic acid	149	78	475
Phthalic acid	2323	5352	1746
Phosphoethanolamine	18588	13310	12940
Phosphoenolpyruvate	724	1509	2098
Phosphate	450729	413717	284875

Average Concentration

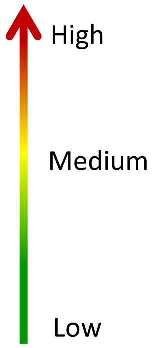

Phenylpyruvate	470	269	2836
Phenylethylamine	1194	1106	1040
Phenylalanine	43240	12760	39466
Pentadecanoic acid	3166	2233	2943
Pelargonic acid	3386	7920	2861
Oxalureate	920	16229	11619
Pantothenic acid	364	268	402
Palmitic acid	141607	100525	164061
Pyroglutamic acid	76439	49054	30312
Oxalic acid	237	1779	1520
Orotic acid	372	93	166
Ornithine	7402	2134	3579
Oleic acid	343	301	378
Octadecanol	385	348	340
Noradrenaline	115	1026	1824
Nonadecanoic acid	1216	3032	1212
Nicotinic acid	11908	6973	17151
Nicotinamide	8211	7332	4994
N-Acetylputrescine	5036	2295	8079
N-Acetylornithine	2992	35055	34778
Acetylglycine	7917	298	667
N-Acetyl-D-galactosamine	247	217	251
N-Acetylaspartic acid	175	93	144
Myristic acid	2911	2118	2576
Myo-Inositol	441	226	170
Methionine sulfoxide	5595	3697	3869
Methionine	94	67	92
Maltotriose	369	178	45
Maltose	3397	2748	1993
Malonic acid	108	1485	1263
Malic acid	1367	2238	3279
Lyxose	2225	1711	1259
Lysine	34834	17970	4741
Leucine	49581	14196	36849
Lauric acid	2474	4844	5963
Lactulose	59	315	395
Lactic acid	1720	1978	1595
Prostaglandin F2a	687	534	416
D-Threitol	191	237	144
Isoribose	500	22714	315
Isoleucine	95305	28855	93882
Inosine	255	238	4244
Indole-3-lactate	103	65	243
Indole-3-acetate	362	251	1449
Hypoxanthine	3000	2606	79935
Hydroxylamine	27195	27345	34609
Homoserine	1840	1211	1494

**Histidine**	3361	1409	929
**Glucose 6-phosphate**	533	221	559
**Heptadecanoic acid**	6910	5389	7624
**Guanosine**	41222	34983	65135
**Guanine**	27986	23973	102215
**Glycolic acid**	2347	6393	11665
**Glycine**	9442	3827	10576
**DL-Glycerol 1-phosphate**	12033	9528	7205
**Galactosylglycerol**	7274	3721	3382
**Glyceric acid**	3478	6892	23655
**Glutathione**	790	331	281
**Glutaric acid**	218	291	403
**Glutamine**	422	574	312
**Glutamic acid**	34449	23015	12208
**Glucose-6-phosphate**	1702	935	1228
**Glucose-1-phosphate**	3806	4357	4466
**Glucose**	827	1166	128
**Galactonic acid**	129	371	128
**Galactinol**	1754	1507	875
**Fumaric acid**	1760	1740	3484
**Fucose**	1125	1034	1152
**Fructose-6-phosphate**	1348	624	990
**Fructose**	519	312	296
**Ethanolamine**	91172	48660	32633
**Dodecanol**	697	478	570
**Dehydroascorbic acid**	284	237	383
**Dehydroabietate**	5068	2649	3403
**Cytosine**	2070	1992	2764
**Cytidine monophosphate**	2092	1380	1567
**Cysteinylglycine**	819	662	588
**Cysteine**	212	80	286
**Conduritol-beta-epoxide**	3554	697	710
**Citrulline**	1643	1016	727
**Citric acid**	3801	5696	5525
**Citramalic acid**	305	317	429
**Citraconic acid**	4068	3815	6766
**Cholesterol**	289	707	345
**Cellobiose**	643	309	75
**Capric acid**	374	373	371
**2-Pyrrolidinone**	454	872	1144
**Beta-Hydroxymyristic acid**	110	89	390
**Beta-Glycerophosphoric acid**	404	270	356
**Gentiobiose**	3860	1729	1849
**Beta-Alanine**	1774	5010	5154
**Benzoic acid**	1311	1047	1240
**Behenic acid**	13791	19539	21304
**Aspartic acid**	12836	8558	4509

Arachidic acid	18807	4359	16100
Aminomalonate	195	196	202
Alpha-Ketoglutarate	35	42	45
Alpha-Aminoadipic acid	81	95	481
Alanine-?-Alanine	451	537	700
D-Alanyl-D-alanine	143	3200	85
Alanine	61574	36702	40065
Adipic acid	1235	443	770
Adenosine monophosphate	15719	11558	12627
Adenosine	132031	122072	158352
Adenine	208809	140678	365902
Acetophenone	3073	2437	3061
7-Methylguanine	159	100	446
Quinovose	753	1465	2483
Glucitol, 6-deoxy-	665	628	525
5-Methoxytryptamine	5015	5581	2852
5'-Methylthioadenosine	402	197	188
5-Aminovaleric acid	1087	680	2169
4-Hydroxyphenylacetic acid	738	688	5965
4-Hydroxybutyric acid	747	115	174
4-Hydroxybenzoate	286	165	668
4-Aminobutanoate	587	457	1233
3-Phosphoglycerate	12164	9697	10100
3-Phenyllactic acid	675	380	4144
3-Hydroxybutyric acid	3742	2000	2987
3'-Adenylic acid	15158	10744	4691
Propanoic acid	887	404	516
2-Ketoisocaproic acid	2241	1323	2248
2-Ketoadipic acid	2916	4345	4569
2-Hydroxyvaleric acid	292	2103	2659
2-Hydroxyhexanoic acid	535	332	2052
D-2-Hydroxyglutaric acid	199	448	954
2,4-Dihydroxybutanoic acid	282	893	936
2,5-Dihydroxypyrazine	3336	1847	799
2,4-Diaminobutyric acid	222	3333	3332
4-Deoxyerythronic acid	37	100	131
1-Monostearin	542	316	357
1-Monopalmitin	6007	3616	2651
1-Deoxyerythritol	131431	113556	37256
1,3-Diaminopropane	2114	1540	1705
704730	1209	1060	873
160962	2786	1578	3427
160842	3212	2118	6928
159824	2485	2450	2227
146957	4025	2854	3467
146262	453	295	362
146042	814	25640	36863

145865	492	620	1945
134760	753	1313	2446
134752	56	276	184
134122	468	319	241
133242	2109	2013	2317
132976	1528	3671	1837
131620	89075	67779	75353
130797	1605	3562	3666
130396	576	994	531
129313	4581	1224	2289
127277	5060	2666	2914
125786	5131	2168	2421
124903	7486	8263	8118
124568	1330	304	709
123989	64	535	465
121002	1814	1555	1405
120789	193	137	266
119066	1060	824	1022
113700	155	230	1121
112264	12541	9117	16407
111826	3075	1769	4350
111057	291	453	122
110359	1001	470	1973
110346	4772	2935	9272
110265	250	824	2392
110131	1682	895	1014
108309	275	143	354
106742	268313	195512	241923
104906	4247	3110	3613
104022	311	498	247
103857	38	57	95
103138	492	328	155
103102	27592	22468	16942
102232	2969	1814	2080
100723	5437	6191	476
88786	848	293	555
88502	3660	2846	3362
88046	1432	848	387
87947	102	108	109
87312	1775	1019	1623
87282	1092	681	2230
84565	1084	817	415
84209	5028	5603	2869
66261	153	1449	2484
48608	50	213	222
47420	3561	2308	2373
47170	886	448	918

46357	1259	10855	9490
46128	736	432	657
41989	91	700	388
41811	819	246	600
41808	988	742	947
33999	84	298	178
32148	128	500	1020
31460	846	1046	1123
31408	1353	1191	1254
31362	3361	1991	5350
31359	1544	1531	1327
31285	2580	2075	6269
26062	1149	775	2570
21885	1509	1264	1233
21683	2565	2121	3332
21666	2847	3750	3306
21665	2263	2450	2288
21664	2981	2406	2700
21511	269	1878	3529
20903	1194	3868	4777
20330	2460	1552	3290
20282	6077	6475	4564
18588	117	48	63
18485	3774	2508	4979
18266	268	143	61
18248	208	202	46
18225	586	2320	591
18177	122	121	385
17962	3364	1657	4617
17830	2125	1619	774
17775	167	393	113
17651	640	221	335
17463	2542	666	375
17437	2570	2191	1879
17245	1559	1447	1361
17068	629	829	912
17002	1663	1486	413
14703	194	473	673
14697	352	169	111
10176	845	787	1403
9320	7135	7350	7682
7408	342	1633	2454
7403	55	3313	3269
5691	228	1146	743
5523	2509	1381	3260
5346	4924	4976	4722
4945	790	677	674

**4937**	1268	864	867
**4850**	41	76	63
**4712**	3872	3581	5565
**4550**	897	998	755
**4265**	2999	2033	3558
**4264**	3093	2103	5221
**4263**	4598	2264	6011
**3188**	860	487	355
**3122**	7571	12008	773
**2847**	1152	874	761
**2706**	2568	1649	2598
**2543**	1253	909	965
**2503**	879	443	230
**2438**	27329	20235	19607
**2262**	4861	7683	5768
**2242**	32198	32955	21957
**2233**	1489	615	387
**2042**	829	1277	7688
**2039**	2215	2309	1714
**2031**	3784	1888	1741
**2030**	2262	1478	2888
**2028**	3025	1411	3237
**2017**	204	373	717
**2001**	1018	2721	1388
**1996**	396	3513	3074
**1981**	2150	1774	1482
**1970**	2227	1137	1067
**1969**	687	421	1545
**1941**	820	898	662
**1912**	1817	1278	1612
**1878**	4857	3849	3807
**1875**	4119	4160	3796
**1872**	5742	4700	4018
**1852**	565	374	1219
**1826**	2443	1031	2788
**1815**	3874	1570	4913
**1812**	657	269	164
**1809**	1709	668	1862
**1806**	2463	1223	4604
**1805**	2206	2055	1531
**1803**	296	155	213
**1799**	6129	73685	41645
**1760**	1030	1180	7663
**1753**	764	945	601
**1751**	2109	1487	1900
**1746**	3406	2280	2195
**1744**	6395	3124	3610

**1737**	2517	2547	226
**1735**	3723	1903	2141
**1725**	3056	3393	3343
**1721**	951	380	451
**1719**	258	145	1272
**1717**	10065	7106	6581
**1713**	6254	2433	3563
**1708**	24102	15134	14031
**1702**	16333	8864	8592
**1701**	3673	1680	1692
**1696**	1031	461	1021
**1675**	372	327	361
**1673**	2209	1275	1278
**1666**	1083	5025	579
**1661**	2431819	1573098	2048030
**1064**	2242	2107	2033
**816**	3243	3100	2590
**453**	35060	9000	29212
**443**	11996	15854	11730
**307**	26220	10259	6099
**257**	1521	867	1210
**168**	1588	636	735
**137**	3819	4746	4407
**134**	14183	4131	5553
**110**	3573	2833	3726
**47**	9326	9167	7576
