# Supplementary material for: A Comparative Analysis of the Metabolomic Response of Electron Beam Inactivated E. coli O26:H11 and Salmonella Typhimurium ATCC 13311
Source: Front Microbiol. 2019 Apr 9;10:694. doi: 10.3389/fmicb.2019.00694 (PMC6465604; doi:10.3389/fmicb.2019.00694)
Supplement: Supplementary file 3 [file Data_Sheet_3.pdf]

**Supplementary Data 3. Differentially Expressed Metabolites in *E. coli* O26:H11**

| Metabolite | EB 0 h – 0 kGy Control | EB 0 h – EB 24 h | 0 kGy Control – EB 24 h | FDR | p value | -LOG10(p) |
|---|---|---|---|---|---|---|
| 48608 | * | | * | 0.0001 | 0.0000 | 6.3887 |
| 4-Hydroxybutyric acid | * | * | | 0.0005 | 0.0000 | 5.5033 |
| 2,4-Diaminobutyric acid | * | | * | 0.0016 | 0.0000 | 4.8192 |
| Malonic acid | * | | * | 0.0018 | 0.0000 | 4.6315 |
| 2,3-Dihydroxybutanedioic acid | * | | * | 0.0031 | 0.0000 | 4.3122 |
| Beta-alanine | * | | * | 0.0040 | 0.0001 | 4.0532 |
| Glucose-6-phosphate | | * | * | 0.0040 | 0.0001 | 4.0328 |
| 2-Hydroxyhexanoic acid | * | * | * | 0.0040 | 0.0001 | 3.9929 |
| 134760 | * | | * | 0.0043 | 0.0001 | 3.9083 |
| Glutaric acid | | * | * | 0.0049 | 0.0002 | 3.7753 |
| N-acetylornithine | * | | * | 0.0049 | 0.0002 | 3.7218 |
| Quinovose | * | | * | 0.0049 | 0.0002 | 3.6850 |
| Xanthosine | | * | * | 0.0049 | 0.0002 | 3.6513 |
| 1702 | * | | * | 0.0049 | 0.0002 | 3.6049 |
| Indole-3-lactate | | * | * | 0.0049 | 0.0002 | 3.6048 |
| Nicotinic acid | | * | * | 0.0049 | 0.0003 | 3.5996 |
| Hydroxyphenyllactic acid | | * | * | 0.0054 | 0.0003 | 3.5364 |
| 3-Phenyllactic acid | | * | * | 0.0073 | 0.0004 | 3.3799 |
| 1799 | * | | * | 0.0100 | 0.0006 | 3.2192 |
| 1996 | * | | * | 0.0116 | 0.0007 | 3.1313 |
| 7403 | * | | * | 0.0137 | 0.0009 | 3.0371 |
| Glycolic acid | * | | * | 0.0137 | 0.0010 | 3.0076 |
| 4850 | * | | * | 0.0137 | 0.0010 | 2.9996 |
| 4-Hydroxyphenylacetic acid | | * | * | 0.0137 | 0.0010 | 2.9791 |
| 1713 | * | | * | 0.0184 | 0.0015 | 2.8331 |
| 1970 | * | | * | 0.0197 | 0.0016 | 2.7869 |
| 18177 | * | * | | 0.0207 | 0.0018 | 2.7490 |
| Citraconic acid | * | | * | 0.0223 | 0.0020 | 2.7019 |
| 108309 | | * | * | 0.0235 | 0.0023 | 2.6350 |
| Indole-3-acetate | | * | * | 0.0235 | 0.0023 | 2.6332 |
| Pinitol | * | | * | 0.0235 | 0.0025 | 2.6106 |
| Succinic acid | | * | * | 0.0235 | 0.0025 | 2.6081 |
| 110131 | * | * | | 0.0235 | 0.0025 | 2.6071 |
| 41989 | * | | * | 0.0253 | 0.0027 | 2.5625 |
| Cysteine | * | | * | 0.0258 | 0.0029 | 2.5417 |
| 31285 | | * | * | 0.0263 | 0.0032 | 2.5008 |
| 5691 | | * | * | 0.0263 | 0.0032 | 2.4983 |
| 5-Aminovaleric acid | | * | * | 0.0263 | 0.0032 | 2.4969 |
| N-acetyl-D-galactosamine | * | | * | 0.0275 | 0.0035 | 2.4572 |
| Fumaric acid | | * | * | 0.0275 | 0.0036 | 2.4461 |
| 124568 | * | | * | 0.0275 | 0.0036 | 2.4432 |
| Phenylpyruvate | | | * | 0.0275 | 0.0037 | 2.4292 |

| | | | | | |
|---|---|---|---|---|---|
| Ethanolamine | | * | * | 0.0275 | 0.0038 | 2.4183 |
| Hypoxanthine | | * | * | 0.0275 | 0.0038 | 2.4146 |
| Isoribose | | * | * | 0.0281 | 0.0041 | 2.3905 |
| 87282 | | * | * | 0.0281 | 0.0041 | 2.3857 |
| 7408 | * | | * | 0.0313 | 0.0047 | 2.3297 |
| Alpha-ketoglutarate | * | * | | 0.0314 | 0.0048 | 2.3188 |
| Pseudouridine | | * | * | 0.0341 | 0.0053 | 2.2743 |
| Propanoic acid | | * | | 0.0371 | 0.0060 | 2.2212 |
| 1760 | * | | * | 0.0371 | 0.0060 | 2.2201 |
| Oxalic acid | * | | * | 0.0380 | 0.0064 | 2.1936 |
| 4-Hydroxybenzoate | | * | * | 0.0380 | 0.0064 | 2.1928 |
| Orotic acid | * | | * | 0.0384 | 0.0067 | 2.1767 |
| Citramalic acid | * | | * | 0.0384 | 0.0069 | 2.1610 |
| Inosine | | * | * | 0.0384 | 0.0069 | 2.1610 |
| 2-Ketoisocaproic acid | * | | * | 0.0384 | 0.0070 | 2.1572 |
| Aspartic acid | | * | * | 0.0401 | 0.0074 | 2.1281 |
| D-2-Hydroxyglutaric acid | | * | | 0.0401 | 0.0075 | 2.1233 |
| Thymine | | | * | 0.0416 | 0.0080 | 2.0983 |
| Cysteinylglycine | * | | * | 0.0416 | 0.0081 | 2.0927 |
| 32148 | | * | * | 0.0468 | 0.0092 | 2.0344 |
| 2031 | * | | * | 0.0471 | 0.0095 | 2.0245 |

*Metabolite has significantly different concentrations between groups
