# Supplementary material for: A Comparative Analysis of the Metabolomic Response of Electron Beam Inactivated E. coli O26:H11 and Salmonella Typhimurium ATCC 13311
Source: Front Microbiol. 2019 Apr 9;10:694. doi: 10.3389/fmicb.2019.00694 (PMC6465604; doi:10.3389/fmicb.2019.00694)
Supplement: Supplementary file 4 [file Data_Sheet_4.pdf]

Supplementary Data 4. Differentially Expressed Metabolites in *S.* Typhimurium

Metabolite	EB 0 h – 0 kGy Control	EB 0 h – EB 24 h	0 kGy Control – EB 24 h	FDR	p.value	-LOG10(p)
Thymine	*	*	*	7.21E-05	2.29E-07	6.6393
Succinic acid	*	*	*	0.0003746	2.98E-06	5.5259
3122		*	*	0.0003746	5.27E-06	5.2782
31285	*	*	*	0.0003746	6.25E-06	5.2038
Hypoxanthine		*	*	0.0003746	9.00E-06	5.046
Oxalureate	*		*	0.0003746	9.93E-06	5.0032
21511	*	*	*	0.0003746	1.02E-05	4.991
Tyrosol		*	*	0.0003746	1.18E-05	4.9286
3-Phenyllactic acid	*	*	*	0.0003746	1.26E-05	4.8988
7403	*		*	0.0003746	1.30E-05	4.8854
N-Acetylornithine	*		*	0.0003746	1.41E-05	4.8515
Oxalic acid	*		*	0.0003746	1.43E-05	4.8442
4-Hydroxybutyric acid	*	*	*	0.0004326	1.79E-05	4.7469
Malonic acid	*		*	0.0006491	2.89E-05	4.5385
Glycolic acid	*	*	*	0.0006689	3.20E-05	4.4955
2-Hydroxyvaleric acid	*		*	0.000677	3.66E-05	4.4365
453	*	*		0.000677	3.67E-05	4.4359
Conduritol-beta-epoxide	*		*	0.0007188	4.12E-05	4.3851
Phenylalanine	*	*		0.0009483	5.74E-05	4.2412
46357	*		*	0.0010682	6.80E-05	4.1673
Glyceric acid	*	*	*	0.0010969	7.34E-05	4.1345
L-Valine	*	*	*	0.0011305	7.92E-05	4.1013
Pseudouridine		*	*	0.0012618	9.24E-05	4.0342
Phenylpyruvate		*	*	0.0012854	9.82E-05	4.0077
123989	*		*	0.0013298	0.0001097	3.9599
1996	*		*	0.0013298	0.0001101	3.9582
Xanthosine	*	*	*	0.0015058	0.0001295	3.8878
Isoleucine	*	*		0.0020557	0.0001833	3.7368
2-Pyrrolidinone	*		*	0.0021509	0.000202	3.6946
2,4-Dihydroxybutanoic acid	*		*	0.0021509	0.0002055	3.6872
17830		*	*	0.0024257	0.0002395	3.6207
1852	*	*	*	0.0026584	0.0002765	3.5583
146042	*		*	0.0026584	0.0002794	3.5538
160842	*	*	*	0.003138	0.0003398	3.4688
110265	*	*	*	0.003913	0.0004362	3.3603
145865		*	*	0.0048798	0.0005719	3.2427
7408	*		*	0.0048798	0.000575	3.2403
2042		*	*	0.0050237	0.000608	3.2161
Leucine	*	*		0.005494	0.0006834	3.1653
88046	*	*	*	0.005494	0.0006999	3.155
Guanine		*	*	0.0059105	0.0007718	3.1125
Trehalose	*	*	*	0.0060039	0.0008031	3.0952

17002		*	*	0.0060981	0.0008351	3.0783
130797	*		*	0.0064164	0.0008991	3.0462
17463	*		*	0.0065385	0.0009371	3.0282
L-Tryptophan	*	*		0.007616	0.0011157	2.9524
Pyrophosphate		*	*	0.0078104	0.0011691	2.9322
47170	*	*		0.0093215	0.0014249	2.8462
2233	*		*	0.0097486	0.0015213	2.8178
Propanoic acid	*		*	0.0098891	0.0015747	2.8028
Aspartic acid		*	*	0.010114	0.0016427	2.7844
Lysine		*	*	0.010276	0.0017229	2.7637
Ethanolamine	*		*	0.010276	0.0017344	2.7608
66261	*		*	0.010382	0.0017854	2.7483
2,4-Diaminobutyric acid	*		*	0.010884	0.0019353	2.7133
Beta-Alanine	*		*	0.010884	0.0019673	2.7061
Dehydroabietate	*		*	0.010884	0.0019757	2.7043
2,3-Dihydroxybutanedioic acid	*		*	0.012613	0.0023298	2.6327
Ornithine	*		*	0.012622	0.0024279	2.6148
D-2-Hydroxyglutaric acid	*	*	*	0.012622	0.0024408	2.6125
Noradrenaline	*		*	0.012622	0.002483	2.605
Galactosylglycerol	*		*	0.012622	0.0024922	2.6034
41989	*		*	0.013524	0.0027134	2.5665
Indole-3-acetate		*	*	0.01378	0.0028087	2.5515
2-Hydroxybenzaldehyde		*	*	0.015544	0.0032177	2.4924
1812	*		*	0.016224	0.0034101	2.4672
110346		*	*	0.017032	0.0036341	2.4396
84209		*	*	0.017164	0.0037171	2.4298
Pinitol	*		*	0.017378	0.0038238	2.4175
Pyroglutamic acid			*	0.017378	0.0039281	2.4058
2,5-Dihydroxypyrazine			*	0.017378	0.0039295	2.4057
2-Hydroxyhexanoic acid		*	*	0.018988	0.0043538	2.3611
4-Hydroxybenzoate		*	*	0.019101	0.0044406	2.3526
88786	*	*		0.019747	0.0046538	2.3322
3'-Adenylic acid		*	*	0.020222	0.0048301	2.316
5-Methoxytryptamine		*	*	0.023904	0.0057857	2.2376
1719		*	*	0.025331	0.0062592	2.2035
Quinovose			*	0.025331	0.0062925	2.2012
1735	*		*	0.025733	0.0064742	2.1888
87282		*		0.029032	0.0074568	2.1274
2503			*	0.029032	0.0074892	2.1256
Myo-Inositol	*		*	0.031351	0.0081872	2.0869
112264		*		0.031365	0.0083831	2.0766
2262	*			0.031365	0.0083906	2.0762
1970	*		*	0.03142	0.0085053	2.0703
32148			*	0.032109	0.0087943	2.0558
1713	*			0.033008	0.0091455	2.0388
84565		*	*	0.035117	0.0098418	2.0069
1969		*		0.037358	0.010589	1.9752

4-Hydroxyphenylacetic acid		*	*	0.038151	0.010935	1.9612
Threonine	*		*	0.039247	0.011374	1.9441
Glucose		*	*	0.040043	0.011802	1.928
Glutamic acid			*	0.040043	0.01186	1.9259
1721	*		*	0.041419	0.012399	1.9066
Citrulline			*	0.045244	0.013689	1.8636
Urocanic acid		*		0.045693	0.01397	1.8548
Alanine	*		*	0.049051	0.015153	1.8195
Inosine		*	*	0.049631	0.01549	1.8099
