# Supplementary material for: A Comparative Analysis of the Metabolomic Response of Electron Beam Inactivated E. coli O26:H11 and Salmonella Typhimurium ATCC 13311
Source: Front Microbiol. 2019 Apr 9;10:694. doi: 10.3389/fmicb.2019.00694 (PMC6465604; doi:10.3389/fmicb.2019.00694)
Supplement: Supplementary file 5 [file Data_Sheet_5.PDF]

Supplementary Data 5. *E. coli* O26:H11 Pathway Analysis: 0 kGy Control – EB 0 h

Metabolic Pathway	Total Compounds	Hits	Raw p value	-log(p)	FDR	Impact
Pentose and glucuronate interconversions	33	4	0.000799	7.1324	0.02038	0.10593
beta-Alanine metabolism	16	7	0.000944	6.9653	0.02038	0.69231
Starch and sucrose metabolism	31	8	0.001308	6.6392	0.02038	0.44291
Pantothenate and CoA biosynthesis	23	5	0.00151	6.4959	0.02038	0.16794
Propanoate metabolism	20	3	0.002479	6	0.02677	0.05405
Sulfur metabolism	13	3	0.00457	5.3883	0.041126	0.06944
Thiamine metabolism	19	2	0.007083	4.95	0.044261	0
Amino sugar and nucleotide sugar metabolism	42	5	0.007411	4.9048	0.044261	0.09561
Galactose metabolism	37	6	0.00762	4.877	0.044261	0.14286
Cysteine and methionine metabolism	34	6	0.008196	4.8041	0.044261	0.22108
Arginine and proline metabolism	41	12	0.009127	4.6965	0.044806	0.4923
Lysine degradation	11	2	0.012038	4.4197	0.052194	0
C5-Branched dibasic acid metabolism	6	1	0.012565	4.3768	0.052194	0
Histidine metabolism	13	1	0.015125	4.1914	0.05834	0.04264
Phenylalanine, tyrosine and tryptophan biosynthesis	23	5	0.019384	3.9433	0.064113	0
Tyrosine metabolism	10	2	0.019513	3.9367	0.064113	0
Valine, leucine and isoleucine biosynthesis	26	6	0.020184	3.9029	0.064113	0.05425
Tryptophan metabolism	11	2	0.021744	3.8284	0.065231	0.2
Purine metabolism	73	12	0.0232	3.7636	0.065938	0.09099
Glycine, serine and threonine metabolism	32	7	0.025842	3.6557	0.066439	0.53438
Novobiocin biosynthesis	3	1	0.026912	3.6152	0.066439	0
Glutathione metabolism	21	8	0.027068	3.6094	0.066439	0.52728
Pyrimidine metabolism	44	9	0.036633	3.3068	0.086008	0.29759
Lysine biosynthesis	13	3	0.041762	3.1758	0.091973	0
Aminoacyl-tRNA biosynthesis	66	18	0.043498	3.135	0.091973	0.13043
Pyruvate metabolism	26	2	0.044284	3.1171	0.091973	0.1077
Glyoxylate and dicarboxylate metabolism	29	4	0.049269	3.0105	0.098537	0.15119
Valine, leucine and isoleucine degradation	23	4	0.051845	2.9595	0.099986	0
Glycerolipid metabolism	14	2	0.057439	2.857	0.10696	0.26087
Alanine, aspartate and glutamate metabolism	18	7	0.059522	2.8214	0.10714	0.90426
Butanoate metabolism	18	4	0.072148	2.629	0.12568	0.05882
Phenylalanine metabolism	23	5	0.10494	2.2543	0.17709	0.00316

Ubiquinone and other terpenoid-quinone biosynthesis	15	1	0.1177	2.1397	0.19259	0
Nitrogen metabolism	18	6	0.12343	2.0921	0.19603	0
Porphyrin and chlorophyll metabolism	33	1	0.1328	2.0189	0.20488	0
Citrate cycle (TCA cycle)	20	5	0.14307	1.9445	0.21004	0.2289
D-Glutamine and D-glutamate metabolism	7	2	0.14392	1.9385	0.21004	0.17241
Cyanoamino acid metabolism	8	3	0.15796	1.8454	0.22447	0
Streptomycin biosynthesis	9	4	0.16265	1.8162	0.22521	0.37143
Methane metabolism	11	2	0.22931	1.4727	0.30957	0.16667
Selenoamino acid metabolism	18	1	0.24615	1.4018	0.3242	0
Fatty acid metabolism	41	1	0.26084	1.3438	0.33537	0
Biosynthesis of unsaturated fatty acids	6	2	0.28312	1.2619	0.35554	0
Benzoate degradation via CoA ligation	10	3	0.33233	1.1016	0.40786	0
Nicotinate and nicotinamide metabolism	13	3	0.34559	1.0625	0.41471	0.14362
Riboflavin metabolism	14	1	0.51634	0.66099	0.58387	0
Glycerophospholipid metabolism	23	2	0.51762	0.65852	0.58387	0.21579
Fructose and mannose metabolism	30	1	0.519	0.65586	0.58387	0
D-Alanine metabolism	3	2	0.59678	0.5162	0.65768	0
Inositol phosphate metabolism	8	1	0.65366	0.42516	0.70596	1
Peptidoglycan biosynthesis	19	3	0.69655	0.36161	0.73753	0.09055
Polyketide sugar unit biosynthesis	5	1	0.74252	0.2977	0.77108	0
Glycolysis or Gluconeogenesis	29	3	0.80795	0.21326	0.82319	0.09195
Pentose phosphate pathway	26	4	0.9059	0.098824	0.9059	0.22822
