# Supplementary material for: A Comparative Analysis of the Metabolomic Response of Electron Beam Inactivated E. coli O26:H11 and Salmonella Typhimurium ATCC 13311
Source: Front Microbiol. 2019 Apr 9;10:694. doi: 10.3389/fmicb.2019.00694 (PMC6465604; doi:10.3389/fmicb.2019.00694)
Supplement: Supplementary file 6 [file Data_Sheet_6.PDF]

Supplementary Data 6. E. coli O26:H11 Pathway Analysis: EB 0 h – EB 24 h

Metabolic Pathway	Total Compounds	Hits	Raw p value	-log(p)	FDR	Impact
Cysteine and methionine metabolism	34	6	0.000227	8.3911	0.006461	0.22108
Citrate cycle (TCA cycle)	20	5	0.000358	7.9347	0.006461	0.2289
Alanine, aspartate and glutamate metabolism	18	7	0.000366	7.9136	0.006461	0.90426
Nicotinate and nicotinamide metabolism	13	3	0.000532	7.5386	0.007051	0.14362
Butanoate metabolism	18	4	0.001223	6.7067	0.01296	0.05882
beta-Alanine metabolism	16	7	0.003106	5.7745	0.027435	0.69231
Purine metabolism	73	13	0.006725	5.0019	0.05092	0.0953
Ubiquinone and other terpenoid-quinone biosynthesis	15	1	0.010122	4.5931	0.064851	0
Lysine biosynthesis	13	3	0.011569	4.4594	0.064851	0
Glycerophospholipid metabolism	23	2	0.012236	4.4034	0.064851	0.21579
Phenylalanine, tyrosine and tryptophan biosynthesis	23	5	0.015868	4.1435	0.068666	0
Propanoate metabolism	20	3	0.016907	4.08	0.068666	0.05405
Benzoate degradation via CoA ligation	10	3	0.017381	4.0524	0.068666	0
Sulfur metabolism	13	3	0.018138	4.0097	0.068666	0.06944
Tyrosine metabolism	10	2	0.029218	3.533	0.095959	0
Pantothenate and CoA biosynthesis	23	5	0.029573	3.5209	0.095959	0.16794
Valine, leucine and isoleucine	26	6	0.030779	3.4809	0.095959	0.05425
Phenylalanine metabolism	23	5	0.033289	3.4025	0.098017	0.00316
C5-Branched dibasic acid metabolism	6	1	0.035916	3.3266	0.10019	0
Valine, leucine and isoleucine degradation	23	4	0.037977	3.2708	0.10064	0
Histidine metabolism	13	1	0.045361	3.0931	0.11413	0.04264
Pyrimidine metabolism	44	8	0.047375	3.0497	0.11413	0.24159
Glutathione metabolism	21	8	0.058077	2.846	0.1262	0.52728
Cyanoamino acid metabolism	8	3	0.058433	2.8399	0.1262	0
Aminoacyl-tRNA biosynthesis	66	18	0.059529	2.8213	0.1262	0.13043
Glyoxylate and dicarboxylate metabolism	29	4	0.078137	2.5493	0.15705	0.15119
Thiamine metabolism	19	2	0.080772	2.5161	0.15705	0
Nitrogen metabolism	18	6	0.082972	2.4893	0.15705	0
Selenoamino acid metabolism	18	1	0.087944	2.4311	0.16073	0
Glycine, serine and threonine metabolism	32	7	0.095876	2.3447	0.16938	0.53438
Riboflavin metabolism	14	1	0.10856	2.2205	0.18111	0
Tryptophan metabolism	11	2	0.10935	2.2132	0.18111	0.2
Pyruvate metabolism	26	2	0.13153	2.0285	0.21125	0.1077

Arginine and proline metabolism	41	12	0.15722	1.8501	0.24507	0.4923
D-Alanine metabolism	3	2	0.17084	1.767	0.2587	0
D-Glutamine and D-glutamate metabolism	7	2	0.20928	1.5641	0.3081	0.17241
Pentose and glucuronate interconversions	33	4	0.2843	1.2577	0.40724	0.10593
Fatty acid metabolism	41	1	0.29719	1.2134	0.4145	0
Glycolysis or Gluconeogenesis	29	3	0.33912	1.0814	0.46086	0.09195
Fructose and mannose metabolism	30	1	0.35397	1.0385	0.46303	0
Biosynthesis of unsaturated fatty acids	6	2	0.35819	1.0267	0.46303	0
Peptidoglycan biosynthesis	19	3	0.38184	0.96274	0.48185	0.09055
Methane metabolism	11	2	0.4001	0.91603	0.49315	0.16667
Polyketide sugar unit biosynthesis	5	1	0.41694	0.8748	0.50223	0
Porphyrin and chlorophyll metabolism	33	1	0.43905	0.82313	0.51711	0
Streptomycin biosynthesis	9	2	0.47449	0.74551	0.54421	0.22857
Starch and sucrose metabolism	31	7	0.4826	0.72857	0.54421	0.44291
Amino sugar and nucleotide sugar metabolism	42	4	0.51962	0.65465	0.57375	0.09561
Novobiocin biosynthesis	3	1	0.53253	0.63012	0.576	0
Pentose phosphate pathway	26	4	0.54495	0.60706	0.57765	0.22822
Glycerolipid metabolism	14	2	0.59451	0.52002	0.61782	0.26087
Galactose metabolism	37	4	0.66701	0.40495	0.67984	0.14286
Lysine degradation	11	2	0.73058	0.31392	0.73058	0
