# Supplementary material for: A Comparative Analysis of the Metabolomic Response of Electron Beam Inactivated E. coli O26:H11 and Salmonella Typhimurium ATCC 13311
Source: Front Microbiol. 2019 Apr 9;10:694. doi: 10.3389/fmicb.2019.00694 (PMC6465604; doi:10.3389/fmicb.2019.00694)
Supplement: Supplementary file 7 [file Data_Sheet_7.PDF]

Supplementary Data 7. *S.* Typhimurium Pathway Analysis: 0 kGy Control – EB 0 h

Metabolic Pathway	Total Compounds	Hits	Raw p value	-log(p)	FDR	Impact
Tryptophan metabolism	11	2	0.000132	8.9309	0.007009	0.2
Propanoate metabolism	20	3	0.00218	6.1284	0.049652	0.05405
Phenylalanine, tyrosine and tryptophan biosynthesis	23	5	0.003574	5.634	0.049652	0
Phenylalanine metabolism	23	5	0.003747	5.5867	0.049652	0.00316
Pantothenate and CoA biosynthesis	23	4	0.006471	5.0404	0.055145	0.16794
Glycine, serine and threonine metabolism	32	7	0.007528	4.8892	0.055145	0.53438
Tyrosine metabolism	10	2	0.007793	4.8545	0.055145	0
Aminoacyl-tRNA biosynthesis	66	16	0.008324	4.7886	0.055145	0.13043
Selenoamino acid metabolism	18	1	0.010608	4.5462	0.056392	0
Sulfur metabolism	13	2	0.010693	4.5382	0.056392	0
Glyoxylate and dicarboxylate metabolism	29	4	0.011704	4.4478	0.056392	0.15119
Valine, leucine and isoleucine degradation	23	4	0.013098	4.3353	0.057363	0
Arginine and proline metabolism	41	12	0.015228	4.1846	0.057363	0.4923
Valine, leucine and isoleucine biosynthesis	26	6	0.01624	4.1203	0.057363	0.05425
Novobiocin biosynthesis	3	1	0.017317	4.0561	0.057363	0
Thiamine metabolism	19	1	0.017317	4.0561	0.057363	0
Glycerophospholipid metabolism	23	2	0.018658	3.9815	0.058169	0.21579
Glycerolipid metabolism	14	2	0.030508	3.4898	0.085166	0.26087
Lysine degradation	11	2	0.031573	3.4555	0.085166	0
Purine metabolism	73	11	0.032138	3.4377	0.085166	0.0763
Glutathione metabolism	21	7	0.03789	3.2731	0.095627	0.52728
Lysine biosynthesis	13	3	0.042072	3.1684	0.09814	0
Inositol phosphate metabolism	8	1	0.042589	3.1562	0.09814	1
Cysteine and methionine metabolism	34	4	0.047279	3.0517	0.10441	0.13017
beta-Alanine metabolism	16	7	0.049741	3.0009	0.10545	0.69231
Cyanoamino acid metabolism	8	3	0.060152	2.8109	0.12262	0
Histidine metabolism	13	1	0.063148	2.7623	0.12396	0.04264
Nitrogen metabolism	18	6	0.069191	2.6709	0.13097	0
Methane metabolism	11	2	0.080122	2.5242	0.14643	0.16667
Benzoate degradation via CoA ligation	10	3	0.11097	2.1985	0.19604	0
Nicotinate and nicotinamide metabolism	13	3	0.12172	2.106	0.2081	0.14362
Citrate cycle (TCA cycle)	20	4	0.15169	1.8859	0.25123	0.18633
Porphyrin and chlorophyll metabolism	33	1	0.16019	1.8314	0.25727	0

Pyruvate metabolism	26	2	0.23941	1.4296	0.37319	0.1077
Fatty acid metabolism	41	1	0.27665	1.285	0.41893	0
Streptomycin biosynthesis	9	4	0.30266	1.1952	0.44558	0.37143
Biosynthesis of unsaturated fatty acids	6	3	0.32637	1.1197	0.45548	0
Alanine, aspartate and glutamate metabolism	18	6	0.32761	1.1159	0.45548	0.90426
Pyrimidine metabolism	44	8	0.33877	1.0824	0.45548	0.24159
Galactose metabolism	37	8	0.34376	1.0678	0.45548	0.20215
Butanoate metabolism	18	4	0.36252	1.0147	0.45976	0.05882
D-Glutamine and D-glutamate metabolism	7	2	0.36596	1.0052	0.45976	0.17241
D-Alanine metabolism	3	2	0.37301	0.98615	0.45976	0
Peptidoglycan biosynthesis	19	3	0.53796	0.61997	0.648	0.09055
Glycolysis or Gluconeogenesis	29	3	0.58431	0.53732	0.68819	0.09195
Starch and sucrose metabolism	31	8	0.60356	0.5049	0.69541	0.44291
Amino sugar and nucleotide sugar metabolism	42	5	0.68642	0.37627	0.77405	0.09561
Pentose phosphate pathway	26	4	0.7045	0.35027	0.77599	0.22822
C5-Branched dibasic acid metabolism	6	1	0.71742	0.33209	0.77599	0
Fructose and mannose metabolism	30	1	0.75919	0.2755	0.80474	0
Riboflavin metabolism	14	1	0.83015	0.18615	0.86271	0
Polyketide sugar unit biosynthesis	5	1	0.87076	0.13838	0.88751	0
Pentose and glucuronate interconversions	33	4	0.96311	0.037586	0.96311	0.10593
