# Supplementary material for: A Comparative Analysis of the Metabolomic Response of Electron Beam Inactivated E. coli O26:H11 and Salmonella Typhimurium ATCC 13311
Source: Front Microbiol. 2019 Apr 9;10:694. doi: 10.3389/fmicb.2019.00694 (PMC6465604; doi:10.3389/fmicb.2019.00694)
Supplement: Supplementary file 8 [file Data_Sheet_8.PDF]

Supplementary Data 8. *S.* Typhimurium Pathway Analysis: EB 0 h – EB 24 h

Metabolic Pathway	Total Compounds	Hits	Raw p value	-log(p)	FDR	Impact
Alanine, aspartate and glutamate metabolism	18	6	4.59E-06	12.292	0.000243	0.90426
Cysteine and methionine metabolism	34	3	0.00013	8.9475	0.001996	0.09918
Glycerolipid metabolism	14	2	0.000137	8.893	0.001996	0.26087
Nicotinate and nicotinamide metabolism	13	3	0.000151	8.8008	0.001996	0.14362
Glyoxylate and dicarboxylate metabolism	29	4	0.000264	8.2394	0.002799	0.15119
Aminoacyl-tRNA biosynthesis	66	16	0.000358	7.9359	0.00316	0.13043
Benzoate degradation via CoA ligation	10	3	0.000562	7.4838	0.004256	0
Arginine and proline metabolism	41	12	0.000738	7.2112	0.004597	0.4923
Phenylalanine metabolism	23	5	0.000781	7.1554	0.004597	0.00316
Butanoate metabolism	18	4	0.000922	6.9888	0.004888	0.05882
Propanoate metabolism	20	3	0.001185	6.7377	0.005711	0.05405
Tyrosine metabolism	10	2	0.001499	6.5028	0.006622	0
Phenylalanine, tyrosine and tryptophan biosynthesis	23	5	0.00218	6.1287	0.008612	0
Pantothenate and CoA biosynthesis	23	4	0.002275	6.0859	0.008612	0.16794
Citrate cycle (TCA cycle)	20	4	0.003845	5.5609	0.013587	0.18633
Purine metabolism	73	12	0.004944	5.3096	0.016377	0.08061
Nitrogen metabolism	18	6	0.00586	5.1397	0.018268	0
Glycine, serine and threonine metabolism	32	7	0.007162	4.9389	0.020211	0.53438
Lysine biosynthesis	13	3	0.007245	4.9274	0.020211	0
Pyrimidine metabolism	44	8	0.010162	4.5891	0.025994	0.24159
Ubiquinone and other terpenoid-quinone biosynthesis	15	1	0.010299	4.5757	0.025994	0
Valine, leucine and isoleucine degradation	23	4	0.012896	4.3509	0.031067	0
Glycolysis or Gluconeogenesis	29	3	0.016225	4.1212	0.037387	0.09195
Valine, leucine and isoleucine biosynthesis	26	6	0.017303	4.0569	0.03821	0.05425
Pentose phosphate pathway	26	4	0.020303	3.897	0.043042	0.22822
Streptomycin biosynthesis	9	3	0.021785	3.8266	0.044407	0.37143
C5-Branched dibasic acid metabolism	6	1	0.022957	3.7741	0.045064	0
Lysine degradation	11	2	0.027524	3.5927	0.0521	0
Glycerophospholipid metabolism	23	2	0.045073	3.0995	0.082375	0.21579
Tryptophan metabolism	11	2	0.049235	3.0111	0.086982	0.2
beta-Alanine metabolism	16	7	0.056711	2.8698	0.096957	0.69231
Cyanoamino acid metabolism	8	3	0.061173	2.794	0.10132	0

Riboflavin metabolism	14	1	0.068498	2.6809	0.11001	0
Amino sugar and nucleotide sugar metabolism	42	5	0.073678	2.608	0.11485	0.09561
Porphyrin and chlorophyll metabolism	33	1	0.10394	2.2639	0.1574	0
Glutathione metabolism	21	7	0.11537	2.1596	0.16985	0.52728
Novobiocin biosynthesis	3	1	0.132	2.0249	0.18411	0
Thiamine metabolism	19	1	0.132	2.0249	0.18411	0
Methane metabolism	11	2	0.14064	1.9616	0.1906	0.16667
D-Glutamine and D-glutamate metabolism	7	2	0.14385	1.939	0.1906	0.17241
Fatty acid metabolism	41	1	0.22562	1.4889	0.28809	0
Biosynthesis of unsaturated fatty acids	6	3	0.2283	1.4771	0.28809	0
Fructose and mannose metabolism	30	1	0.28594	1.252	0.35244	0
Starch and sucrose metabolism	31	7	0.29891	1.2076	0.36005	0.4084
Sulfur metabolism	13	2	0.3364	1.0894	0.39621	0
Pyruvate metabolism	26	2	0.35868	1.0253	0.41326	0.1077
D-Alanine metabolism	3	2	0.38986	0.94196	0.43963	0
Galactose metabolism	37	6	0.40429	0.90563	0.4464	0.20215
Peptidoglycan biosynthesis	19	3	0.4177	0.87299	0.4518	0.09055
Histidine metabolism	13	1	0.43884	0.82361	0.46518	0.04264
Polyketide sugar unit biosynthesis	5	1	0.5616	0.57696	0.58362	0
Selenoamino acid metabolism	18	1	0.58308	0.53944	0.59429	0
Pentose and glucuronate interconversions	33	4	0.63519	0.45383	0.63519	0.10593
